# Energetic Polyoxetanes as High-Performance Binders for Energetic Composites: A Critical Review

**DOI:** 10.3390/polym14214651

**Published:** 2022-11-01

**Authors:** Kinga Lysien, Agnieszka Stolarczyk, Tomasz Jarosz

**Affiliations:** Department of Physical Chemistry and Technology of Polymers, Silesian University of Technology, 9 Strzody Street, 44-100 Gliwice, Poland

**Keywords:** energetic binder, oxetane, propellant, BAMO, AMMO, NIMMO, energetic polymer, glycidyl azide polymer

## Abstract

Energetic oxetanes, a group of energetic binders (EBs), are the focus of this review. We briefly introduce the role of binders and the difference between EBs and traditional “non-energetic” polymer binders, followed by a discussion of the synthesis and key properties of polyoxetanes. Priority is given to recent works, but a long-term perspective is provided where necessary, to illustrate the development of this field and the most relevant emerging trends. New reports on methods of obtaining oxetane polymers are presented; concerning the possibility of using a new catalyst, water: Al(C_4_H_9_)_3_, or the ratio of comonomers on the properties of the obtained binders. The synthesis of copolymers with the use of polymers with an oxetane ring and polyethers, polybutadiene terminated with hydroxyl groups and poly (3-difluoroaminomethyl-3-methyloxetane) is discussed. The latest developments in crosslinking reactions and crosslinking agents used are also described. The primary challenges faced by the field are identified and a perspective on the future development of polyoxetane EBs is presented.

## 1. Introduction

**Figure 1:**
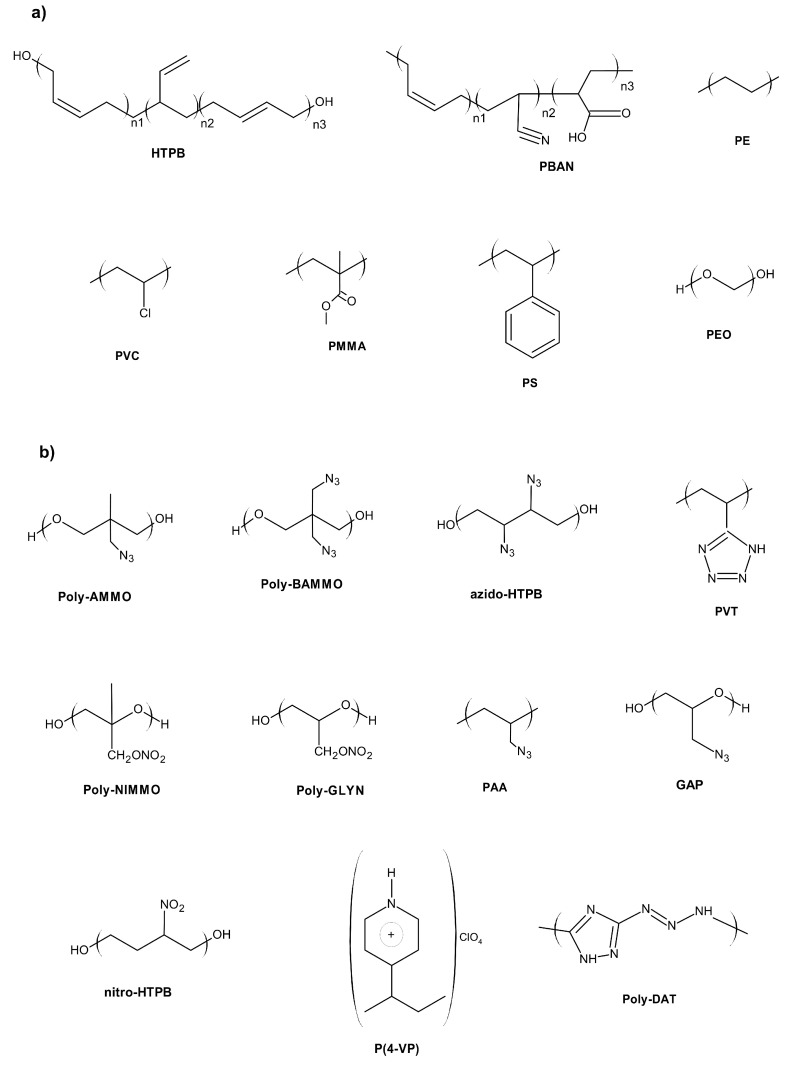
Chemical structures of the most commonly used binders: (**a**) non-energetic; (**b**) energetic binders.

The role of binder in regards to energetic material formulations is to promote cohesion between the components of a formulation and to grant the bound formulation the desired mechanical properties (e.g., flexibility, tensile strength) [1]. Among various types of energetic materials, binders have found the greatest application in solid propellant and polymer-bonded explosive formulations [2,3]. A fairly narrow set of polymers (Figure 1a) is used as binders for the majority of solid propellant and polymer-bonded explosive formulations, despite the two types of energetic materials differing sharply in terms of their properties [4,5].

The repeat units of such traditional polymeric binders are rich in carbon atoms, while generally containing either few or no heteroatoms, such as oxygen and nitrogen. This translates into high demand for oxygen during combustion of the energetic material, necessitating the use of a sufficient amount of oxidising agent so as to avoid compromising the performance of the energetic material. Most oxidising agents, however, decompose endothermally, so increasing their share in the energetic material formulations limits the amount of heat that can be released upon their combustion. Additional energy is often lost on inducing the thermal decomposition of the binders, as most of them exhibit limited reactivity with oxygen.

To alleviate the above issues, a broad category of compounds, denoted as “energetic binders” (EBs), has been developed and was initially represented by a variety of nitropolymers [6] and polyvinylpyridinium perchlorates [7]. Although no definition was given in the initial works on the subject, EBs were intended to allow the development of propellants requiring reduced oxidising agent contents, thereby affording them improved specific impulse values.

Although polyvinylpyridine derivatives and nitropolymers have found wide application as high-performance binders, over the years a number of polymers have been referred to as “energetic binders” (Figure 1). The common structural features present in those systems include functionalisation (nitro-, nitrate- and azido- substituents are most commonly encountered) and replacement of the carbon atoms in the repeat units with oxygen or nitrogen atoms [8]. Among these polymers, particular research attention has recently been dedicated to polyoxetanes.

Energetic polyoxetanes, and particularly those bearing azide functionalities, are increasingly being identified as promising new EBs [9,10,11]. The polyoxetane backbone is relatively flexible, but this parameter can be readily modified by functionalisation. This is well-exemplified by the comparison between AMMO and BAMO, in which case replacing the methyl substituent of the oxetane ring in AMMO with an azidomethyl group (BAMO) significantly alters the rigidity of the resulting polymers [12]. This functional group substitution also manifests as a change in the thermomechanical properties of the polymers, as the glass transition temperatures (T_G_) of poly(AMMO) and poly(BAMO) are −42 °C and −41 °C respectively [13,14,15], compared with a T_G_ of −70 °C observed for hydroxyl-terminated polybutadiene (HTPB). The inclusion of azide functionalities, however, is beneficial from an energetic standpoint, as they readily decompose, releasing both significant amounts of energy and molecular nitrogen [16,17].

Currently, research on energetic polyoxetanes is primarily focused on poly(BAMO) and poly(AMMO). Among this research, developments focusing on reducing the T_G_, so as to ensure that the bound formulations maintain sufficient mechanical parameters in varying temperatures. On the other hand, the introduction of a non-energetic component contributes to limiting the energetic characteristics of any energetic material formulations [18,19,20,21].

**Figure 2:**
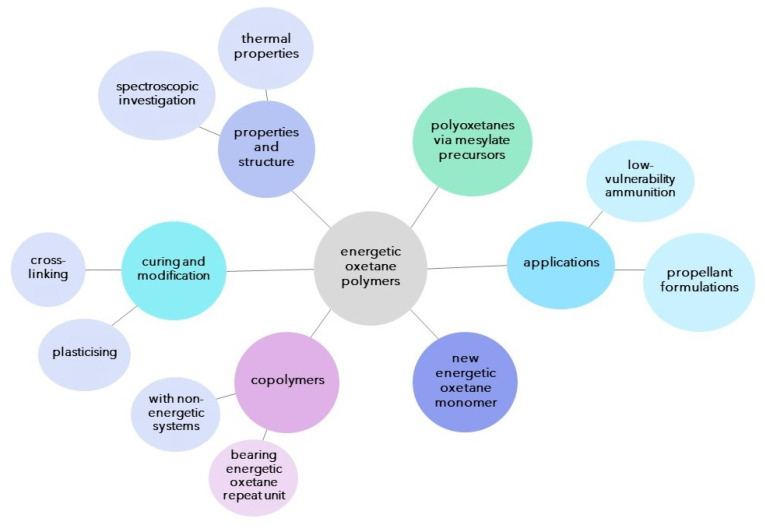
Schematic diagram covering the overall review article.

In this work, we have aimed at summarising the most relevant works on energetic polyoxetanes, discussing their synthesis, curing and most relevant properties (Figure 2). Although energetic polyoxetanes are not as diverse as GAP in their scope of application, their use as energetic binders (EBs) for composite solid propellants. Although the majority of recent reports focus on the synthesis of energetic oxetane copolymers and on modifying these systems via curing or functionalisation, a number of worthwhile efforts are also being undertaken, particularly in regards to the study of decomposition mechanisms of these EBs.

A number of challenges, however, need to be addressed, in terms of both methodology and scope of material investigations. In regards to the former, the use of divergent methods to evaluate EBs greatly hinders comparison of newly reported results with those already existing in literature. This issue is well-exemplified by the reported decomposition temperatures of poly(NIMMO) varying from 129 °C to above 220 °C [22,23] and is even further complicated when investigations include polymers treated with various curing agents. In regards to the latter, many reported investigations are fragmentary—frequently only the synthesis and fundamental properties of the obtained polyoxetane EBs are described, lacking a follow-up description of the mechanical and energetic performance of those EBs, particularly in regards to specific applications (e.g., performance of newly reported EBs in a solid propellant formulation and comparison against the performance of established binders).

## 2. New Trends in the Synthesis of Energetic Oxetane Polymers

**Figure 3:**
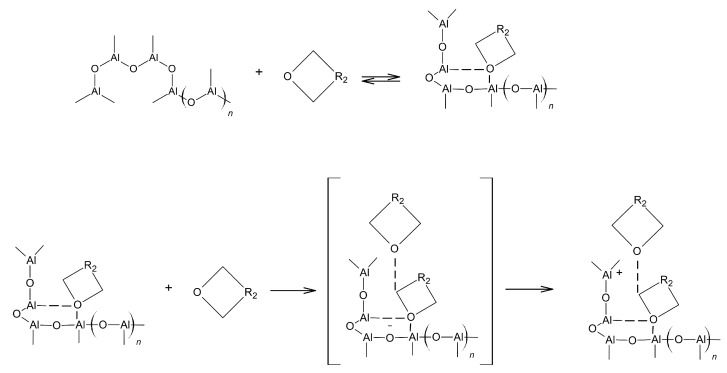
Mechanism of the initial reaction steps for the aluminium-catalysed ring opening polymerisation of oxetanes.

Polymerisation of 3,3-bis(azidomethyl)oxetane (BAMO) and 3-azidomethyl-3-meth-yloxetane (AMMO) proceeds according to the mechanism of ionic/ionic coordination polymerisation. Suitable catalysts for such a reaction are mostly proton donors, usually strong protic (H_2_SO_4_) or aprotic acids (Lewis acid). For the first time a triisobutylaluminium–water (TIBA–water) was used as a catalyst system (Figure 3) [24].

The water:Al(C_4_H_9_)_3_ ratio determines the quantitative composition of the reaction products. In the polymerisation reactions of monomers with electron-donor substituents, catalysts with electron-acceptor properties are used. The catalytic process proceeds through the formation of a catalyst-catalyst complex. The resulting complex acts as a strong acid that initiates polymerisation.

Depending on the catalyst used, BAMO and AMMO polymerisation reactions lead to oligomers with molecular weights on the order of 500–5000 g/mol, when utilising BF_3_ diethyl ether adduct, or on the order of 2 × 10^5^–1 × 10^6^ g/mol, when using triisobutylaluminium.

Monomers with explosophoric groups (azido, nitro) were obtained by substitution of halogeno- or tosyl-substituted compound. Carbon-substituent binding energy decreases in the order [kcal/mol]: C–Cl 91.0, C–Br 81.0, C–I 61.8, C–NO_2_ 37.7; C–N–NO_2_ 58.7; C–SO_2_H_4_C_6_CH_3_ 105.3.

**Figure 4:**
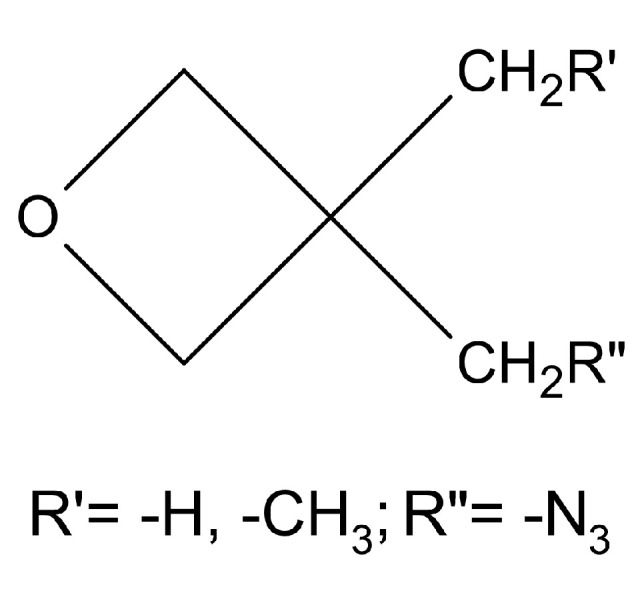
General chemical structure of oxetanes.

**Figure 5:**
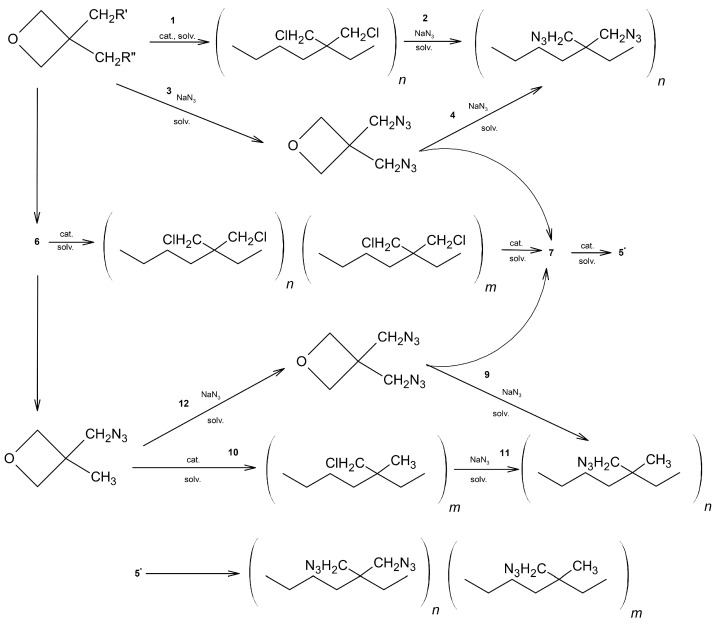
Schematic representation of the synthesis of BAMO and AMMO (monomers) and their polymers and copolymers.

Oxetane ring-containing compounds were polymerised, with a general structure (Figure 4):

The authors performed both direct polymerisation of azido oxetane monomers and also nucleophilic substitution of the corresponding monomers containing a chloride group Figure 5. An important aspect during polymerisation was the maintenance of appropriate conditions. The reactions should be carried out without oxygen and moisture, and only pure monomers should be used. The reactions were carried out in methylene chloride, in the argon atmospheres. The monomer concentrations used were in the range were 2.0–2.5 M.

TIBA-water catalytic system was used at a molar ratio of 1.0:0.8. The use of such a cocatalyst makes it possible to obtain high-molecular polymers with good yields.

The synthesis of poly(AMMO) follows the mechanism of cationic polymerisation of the AMMO monomer [14,25]. For this reason, new routes for the synthesis of AMMO are being sought, bypassing the preparation of the energetic monomer.

### 2.1. Synthesis of Polyoxetanes via Mesylate Precursors

In the synthesis of energetic polymers, the azide group is most often introduced by substitution of halogen functionalities of pre-polymers, such as polyepichlorohydrin in the synthesis of GAP. The substitution reaction itself follows the S_N_2 mechanism, hence, its kinetics are strongly dependent on the chemical properties of the leaving group and the conditions, under which the reaction is carried out. Although halogens are thre most common leaving group, due to the commonly available resources for the ring opening polymerisation of oxetanes and oxiranes, they are a sub-optimal choice of leaving group. The azidation of halogen functionalities is relatively slow, typically requiring very long reaction times to achieve high levels of azidation. Limiting reaction times is not viable, as if azidation is completed at lower conversions, significant amounts of the halogen atoms remain within the “energetic” polymer. Due to their non-flammability, such residual halogen atoms can significantly reduce the energetic performance of the azidated polymer.

Consequently, for high-performance binders, the halogen functionalities are instead replaced with tosylate groups, which are readily substituted in the azidation reaction. However, due to their composition, tosylates induce significant steric congestion, potentially complicating the polymerisation and substitution reactions, particularly in the case of monomers, whose repeat units are comparable in size with this leaving group.

Recently, the use of mesylates has become more common, as they offer all the advantages of tosylates as leaving groups, but are much less bulky and relatively less cost-intensive reagents are necessary to produce mesylate-functionalised monomers than tosylate-functionalised monomers.

The use of this synthetic approach for poly(AMMO) and GAP was compared against the established use of tosylates [26]. Azidation of both the mesyl and tosyl intermediates was reported to proceed quantitatively in the case of both polymers. The reported mesylate-mediated azidation reactions were initially carried out at 75, 85, and 95 °C. When both tosyl and mesyl groups were used, the azide reaction proceeded faster for GAP. Therefore, the range of temperatures, in which the azidation reaction for synthesising poly(AMMO) was increased to 85, 105, and 125 °C, respectively. As expected, a large effect of temperature on the azidation process was observed.

The proposed safer synthesis of poly(AMMO) proceeds in two steps [27]. The first step involves cationic ring-opening polymerisation of 3-mesyloxy-methyl-3-methyl oxetane—a mesylate precursor. The second synthesis step involved an azidation reaction, which was carried out by two methods: homogeneous and phase-transfer catalysis. Phase-transfer catalysis is reported as a more efficient method, as it required only 18 h to achieve 100% conversion, whereas the homogeneous method required as much as 42 h to achieve this degree of conversion.

### 2.2. Development of New Energetic Oxetane Monomers

**Figure 6:**
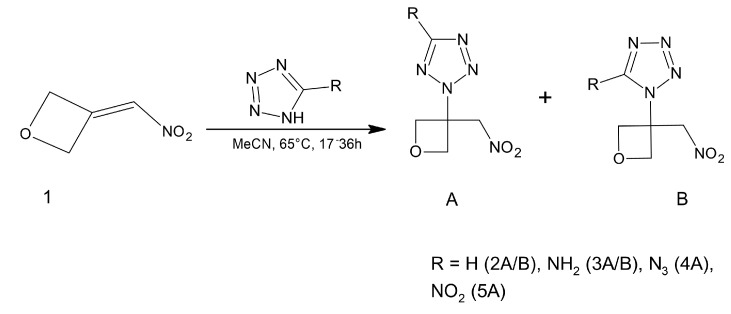
Schematic representation of the synthesis of new energetic oxetane monomers from 3-(nitromethylene)oxetane.

3-(nitromethylene)oxetane was chosen as a precursor for the synthesis of new monomers with potential use as high-energy materials Figure 6.

Tetrazole compounds play a large role in energy materials. This is due to their high nitrogen content and, consequently, positive enthalpy of formation [28]. In addition, the presence of an aromatic ring improves tetrazole stability [28,29].

## 3. Copolymers Bearing Energetic Oxetane Repeat Units

### 3.1. Copolymers of Energetic Oxetanes with Energetic Systems

BAMO-AMMO copolymers were synthesised from different molar ratios to obtain a copolymer with desired properties relevant to high-energy materials [30]. The synthesis was carried out with the following molar ratio of monomers (BAMO:AMMO): 80:20, 50:50, 20:80 (Table 1).

The obtained copolymers were characterised in terms of the typical properties of such compounds.

DSC analysis was used to determine the thermal stability of the copolymers. The DSC plot shows a peak in the vicinity of 252 °C, which corresponds to a Tmax close to poly(BAMO), which accounts for the decomposition of the methyl azide group. Thermogravimetric analysis showed that the decomposition of the copolymers occurs with a three-step mass loss, with the first step being the cleavage of the azide group.

The copolymers were also characterised in terms of rheological properties. An important factor affecting these properties is the crystalline nature of poly(BAMO).

poly(BAMO) is characterised by a high elastic modulus, compared to the viscosity modulus, due to its symmetrical structure. Initially, an increase in viscosity is observed at a higher frequency or shear rate, indicating a shear-thinning nature, followed by a diluent-like behavior. This is likely due to the separation of the polymer structure, making the azide groups more resistant to flow, leading to an increase in viscosity. Similar observations were made for the BAMO-AMMO copolymer (80:20). For the other copolymers, as the AMMO content increases, the viscosity decreases.

The BAMO-AMMO copolymer (80:20) synthesised in the work has the most favorable properties, making it a suitable high-energy material for use in rocket fuel and explosives. It exhibits higher viscosity, compared to elasticity at lower shear rates. It has a wide LVER area, which facilitates mixing of material components.

**Figure 7:**
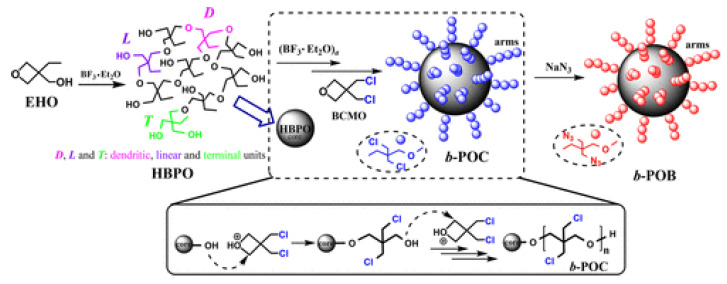
Schematic synthetic route of b-POB. Reprinted with permission from [31]. Copyright 2022 American Chemical Society.

An interesting concept of high energy copolymers is presented in [31]. The authors synthesise a series of star azide copolymers (b-POBs) with hyperbranched polyether cores (HBPO-c) and short linear poly(3,3’-bis-azidomethyl oxetane) arms (poly(BAMO)-a). Schematic route of this synthesises is presented in Figure 7.

The molecular weight of b-POBs increases with the feeding ratio, and that of b-POB-16 can reach 31.7 kg·mol^−1^, displayed relatively low viscosity (liquid state) and T_G_, the values for b-POB-4 achieving the minimum of 5.8 Pa·s (60 °C) and −38 °C, respectively, in spite of its BAMO content reaching to ca. 75.6% (far above that of common linear counterparts). The results from pyrolysis experiments revealed that all b-POB materials had excellent resistance to pyrolysis up to 230 °C (T5%). The enthalpy of formation (up to 2.01 kJ·g^−1^ for b-POB-16) together with high azide content and high heats of decomposition of b-POBs demonstrated their remarkable energy level. The synthetic route of random copolymers poly(GAP-BAMO) with composition 75/25 was presented in [21]. The process start with the synthesis of the halogenated polymeric precursor and then by azidation of the same. The introduction of BAMO units in the GAP chain has the advantage of increasing the number of azide groups and, consequently, the energetic content of the material. This can be done until the amorphous morphology of the polymer is preserved. The operating conditions and the catalytic system were chosen in order to favour a living character of the polymerization and the formation of hydroxyl-terminated chains. The decomposition temperature of such material is 240 °C, thus corresponding to decomposition temperature of GAP. The impact sensitivities of copolymers are only marginally higher than for GAP, but the friction sensitivity is significantly increased from >360 to 288 N. Nevertheless, the copolymers show sensitivity data that are in the typical range of secondary explosive material and can be handled with the common safety precautions.

In the same synthesis way poly(GAP-BAMO) copolymer is described in [32]. The T_G_ of the obtained polymer was determined at −35 °C. DSC curve is due to the second mass loss region between 250 °C and 350 °C. Activation energy calculated at the first region is between α = 0.03 and α = 0.36 with E_a_ of ≈145 kJ/mol and the second region with E_a_ of ≈220 kJ/mol between α = 0.62 and α = 0.8.

The poly(3-azidomethyl-3-methyloxetane) and its copolymers with poly(AMMO) were synthesised by cationic polymerization from 3-tosyloxymethyl-3-methyl oxetane and 3,3-bis(bromomethyl)oxetane, using a polyol as initiator and BFEE as catalyst, followed by azidation. In presence of a BFEE complex and a polyol, cationic homo-and copolymerization of 3-tosyloxymethyl-3-methyl oxetane leads to hydroxyl-terminated polyoxetanes [33].

BAMO–HTPB–BAMO copolymer was synthesised (hydroxyl-terminated polybutadiene (HTPB)). The reaction proceeded via an activated monomer mechanism in which hydroxyl-terminated butadiene acted as a bifunctional diol initiator and BFEE acted as catalyst. The monomer BAMO was added on both sides of the diol initiator, leading to the formation of a triblock copolymer (BAMO–HTPB–BAMO copolymer with a monomer ratio of 17:1). The TGA analysis of copolymer shows a two-stage decomposition process: 223 °C decomposition of the methyl azide of BAMO, and the second at 375 °C decomposition of HTPB and the polymer backbone of BAM. Rheological properties at 40 °C show initially non-Newtonian behavior up to approximately 300 Pa of stress, but afterwards, it becomes almost constant; as the stress increases, the viscosity remains constant around 600 Pa·s [18].

**Figure 8:**
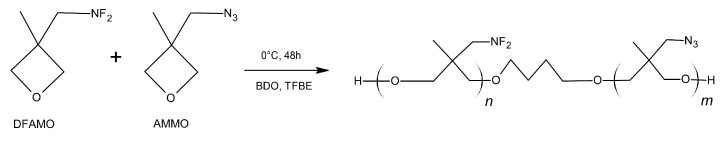
Schematic representation of the synthesis of p(DFAMO-AMMO).

Poly(DFAMO/AMMO copolymer was synthesised, with a good yield (83%) in cationic polymerisation of DFAMO and AMMO (Figure 8) [34].

The synthesis proceeds in 1,2-dichloroethane using TFBE and BDO as catalyst and initiator.

Referring to the proposed mechanism of thermal decomposition of PDA (p(DFAMO-AMMO), the process proceeds in three stages. First, the energy groups of the copolymer are decomposed: difluoroamino and azido. The third mass loss step corresponds to the decomposition of rest of the polymer chain.

DSC method was used to determine the compatibility of the high-energy polymer, with other contact materials. The method used was to determine ΔT_p_ between the contact material and the mixture of contact material and polymer. If ΔT_p_ less than or equal to 2 °C, it is concluded that the materials are compatible.

Referring to the results, PDA is compatible with materials such as RDX, TNT, TATB, PETN. Good compatibility was also found for the standard oxidants AP, AN, KN. Aluminum as a metallic fuel was also found to be compatible with PDA, unlike Mg.

The same authors synthesised DFAMO via multistep raction bromination, cyclization, ammonification, amino protection and fluorination with a total yield of 43% and GC purity of 96% in next step the PDFAMO was obtain by cationic ring-opening polymerisation using boron trifluoride diethyl ether adduct (BFEE) as Lewis acid catalyst, 1,4-butanediol (BDO) as initiator and 1,2-dichloroethane (DCE) as aprotic solvent [35]. The molecular weight (Mn) was about 3000. Auhors provided IR, 1H NMR and 19F NMR. However, the interpretation of the 16F NMR spectra for the polymer is ambiguous.

Poly(3-difluoroaminomethyl-3-methyloxetane/3-nitratomethyl-3-methyloxetane) (Poly-DFAMO-NIMMO) was synthesised according to the cationic polymerisation mechanism, with a good yield of 80% [36]. A copolymer containing both difluoroamine and nitroester groups in its structure may be a promising candidate for use as a high-energy binder. Many scientific publications describe the synthesis or characterization of the properties of DFAMO [37,38] and NIMMO [39,40,41]. Both DFAMO and NIMMO are characterised by their respective good thermal properties and low glass transition temperatures, so obtaining a copolymer containing segments from both polymers in the structure may make it interesting from the point of view of new energy materials.

Compatibility tests determined that PDN appear to be compatible with typical energetic components: RDX, HMX, TATB, PETN, AP, KNO_3_, Al, C1, C.B, Al_2_O_3_ and PbCO_3_. On the other hand, mixing with AN and Mg sensitizes it a bit, while mixing with CL-20, NQ, TNT, NC and NDPA sensitizes it. In comparison, poly(NIMMO) itself was found to be incompatible with CL-20 as per STANAG 4147 criteria [42].

Statistical copolymers of BAMO and NIMMO, as well as BAMO, NIMMO and a polyester have been synthesised [20]. The two types of binders were investigated in a propellant formulation with ammonium perchlorate (23 wt. % binder, 77 wt. % ammonium perchlorate) and found to exhibit moderately good mechanical properties, with the polyester-containing system showing a tensile strength that was slightly lower than the tensile strength of the BAMO-NIMMO copolymer at low temperatures, but exceeded it slightly at higher temperatures, hinting at the different operating temperature ranges for the two systems.

The properties of a hypothetical diblock copolymer of BAMO and NIMMO were predicted theoretically, indicating that depending on the length of the BAMO block, the copolymer can transition between the smectic and disoredered phases or between the nematic, smectic and disordered phases [43]. Changing the BAMO/NIMMO repeat unit ratio was also predicted to allow fine-tuning the fluidity parameters of the copolymer.

Triblock copolymers of NIMMO (core block) and BAMO (outer blocks) were produced using p-bis(α,α-dimethylchloromethyl)benzene and silver hexafluoroantimonate as a non-standard initiating system [44]. The resultant copolymer was found to exhibit a decomposition of 204 °C.

An interesting copolymer of two energetic binders—glycidyl nitrate and NIMMO is reported and compared to the two parent homopolymers [45]. Although the presented data are largely limited, the copolymer is shown to have very similar thermal stability to both parent homopolymers, but exhibits a lower glass temperature, i.e., −37.9 °C as compared to −35.6 °C and −30.2 °C for poly(glycidyl nitrate) and poly(NIMMO) respectively.

P(BAMO-AMMO) copolymer can be sythesised in two ways. The first method involves living polymerisation, while the second involves obtaining poly(BAMO) and poly(AMMO) terminated with diisocyanate groups, and then reacted with a chain extender to obtain a block polymer [46].

**Figure 9:**
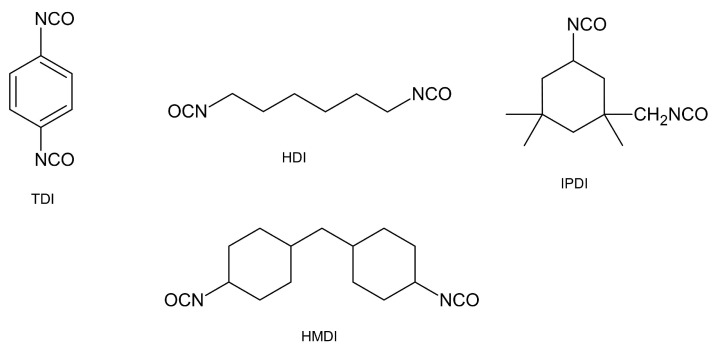
Chemical structure of diisocyanate curing agents investigated in [46].

The effects of different isocyanates on the properties and, in particular, the performance of BAMO-AMMO copolymers were investigated [46]. TDI, HDI, IPDI and HMDI were used (Figure 9), which are typical cross-linking agents that are used to impart appropriate mechanical properties to polymers.

In order to determine the mechanical properties of the obtained samples, the maximum stress σ_m_, as well as breaking elongation ε _b_ were determined (Table 2).

TDI-based polymers had the highest values of maximum stress and breaking elongation. This is due to the higher stiffness and higher cohesion energy of the hard segment. This results in stronger interactions between the polymer chains, and thus to stronger hydrogen bonding between the hard segments. TDI-based polymers have better mechanical properties than the others, and this may indicate their advantage in the propellants field.

The heat of formation was also determined with the group additivity method and heat of combustion method. Again, the highest value (3.75 kJ/g) was obtained for polymers based on the TDI, which can lead to higher energy performance relevant to rocket propellants.

### 3.2. Copolymers of Energetic Oxetanes with Non-Energetic Systems

To compensate for the poor mechanical properties of BAMO, its copolymers with THF are often obtained [47]. Such a solution significantly improves the flexibility of the chain, and thus the mechanical properties. The literature describes the synthesis of BAMO-THF-BAMO triblock copolymer by living polymerisation. Although the mechanical properties of the resulting copolymer were improved, the synthesis itself was complicated [19,48,49,50]. Bis-(chloromethyl) Oxetane copolymer with THF was described by [51]. The synthetic route is also complicated author provided DSC of Bis-(chloromethyl) Oxetane–THF copolymer showed exothermic decomposition with T max at 371 °C and aT_G_ of −30 °C.

Another common solution is to enrich the fuel composition with cross-linking agents. The addition of a small amount of cross-linking agent (<1% by weight), containing a polar group, e.g., a cyano group, leads to the formation of a thin layer of binder particles on the surface, which, by generating chemical or physical bonds between binder and filler molecules, promote interfacial adhesion, thereby affecting mechanical properties.

In order to determine the performance properties of energetic thermoplastic polyurethane elastomers, they were synthesised using various diols: : diethyl bis(hydroxymethyl)malonate (DBM); 2-cyanoethyl-bis(2-hydroxyethyl)amine (CBA) and dicyclohexylmethane-4,4-diiso-cyanate (HMDI). The synthesised polymers contained 0%, 25%, 50%, 75% and 100% of DBM or CBA.

As the DBM or CBA content increased, the maximum tensile strength and the increase in elongation at break decreased. This is due to the presence of side groups in DBM and CBA, the number of which increased in the hard segments and the weakening of hydrogen bonding interactions. As a result, there was a delay in aggregation of hard segments, reducing maximum tensile strength.

Copolymers of BAMO and tetrahydrofuran were produced in both linear and cross-linked (via a standard diisocyanate agent), in order to obtain a binder that is liquid, unlike the solid BAMO [13]. Combustion experiments revealed that the burning rate of the copolymers increased sharply when the fraction of BAMO repeat units in the copolymers was increased. As such, the BAMO content in the copolymers should be increased as much as possible without losing the advantage of the copolymers being liquids.

A triblock copolymer consisting of HTPB, as a non-energetic binder core and two outer blocks of poly(NIMMO) as an energetic binder, was recently prepared [52] and found to have thermal stability very similar to that of poly(NIMMO), while exhibiting a very low glass transition temperature of −76 °C.

The synthesis of a copolymer of NIMMO and THF is reported [53]. The copolymer was then functionalised with terminal vinyl groups and used to produce a polyisoxazoline elastomer. This elastomer is compared with one produced from the copolymer using the traditional isocyanate-curing methodology, employing hexamethylene diisocyanate (HDI), achieving tensile strengths of 1.0 MPa and 3.0 MPa, as well as elongation at break values of 135 % and 300 % respectively for the HDI-cured and isoxazoline-based elastomers. The isoxazoline elastomer was investigated by DSC and found to be compatible with some typical explosives.

A triblock copolymer of NIMMO with a polyether (NIMMO-polyether-NIMMO) has been synthesised [54], but only limited information relevant to its application as an energetic binder are provided. The thermal stability of the copolymer is similar to that of poly(NIMMO) and although the exothermic decomposition of the copolymer was not compared to that of poly(NIMMO), the observation that the heat of this decomposition increases with NIMMO content in the copolymer, is indicative that the polyether segments serve to lower the energetic performance of the copolymer.

## 4. Curing and Modification of Energetic Oxetane Polymers and Copolymers

Organic polymers acting as binders in solid rocket propellants are subjected to cross-linking reactions to give the propellant grains appropriate mechanical properties. Currently, solid rocket propellant formulations include polymers terminated with hydroxyl groups, which react with isocyanates to lead to cross-linked polyurethanes with suitable properties (including high abrasion resistance and high chemical resistance) [55,56,57,58].

Polymers containing azide groups in their structure react with alkynes, according to the Huisgen reaction mechanism, resulting in the formation of triazoles [59,60].

The cross-linking reactions leading to the formation of triazoles are not sensitive to moisture, so there is no need for further process steps to enable the reaction to be carried out under special conditions.

**Figure 10:**
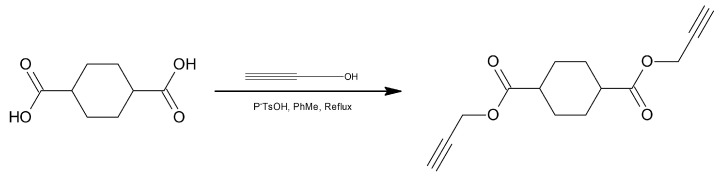
Schematic representation of the synthesis of BPHA.

The mechanical properties and cross-linking reaction kinetics of polymers based on poly(AMMO) were studied [24]. In order to compare the results, GAP-based samples were also examined. Bis-propargyl-1,4-cyclohexyl-dicarboxylate (BPHA) was used as a cross-linking agent. The compound was obtained by mixing cyclohexane-1,4-dicarboxylic acid, propargyl alcohol and p-toluenesulfonic acid in toluene. The reaction was continued for 12 h (Figure 10).

The cross-linking agent and polymer were mixed together at different molar ratios of azide and alkyne groups. The ratios were chosen to obtain densities close to those of typical polyurethanes.

**Figure 11:**
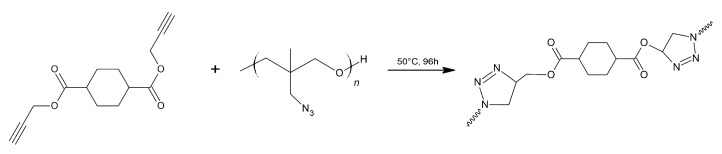
Schematic representation of cross-linking poly(AMMO) using BPHA.

The cross-linking agent and polymer were mixed together at molar ratios (R) of respectively: 1.2, 1.4, 1.6, 1.8, and 2.0. For example, 16.086 g of poly(AMMO) (0.12 mol azide) was mixed with 1.452 g of BPHA. The resulting mixture was left for 96 h at 50 °C to obtain a cross-linked structure (Figure 11).

**Figure 12:**
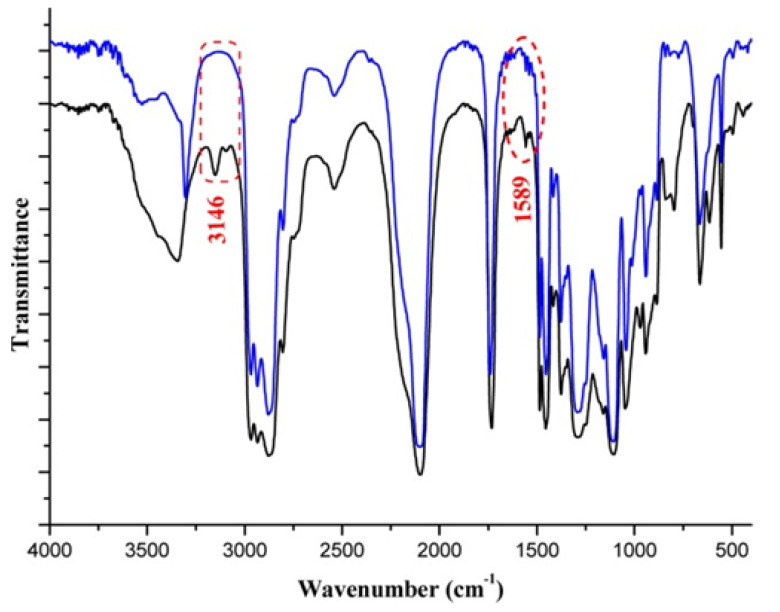
FT-IR spectrum of cross-linked polymer (blue line) and blend of poly(AMMO) and BPHA (black line). Reprinted with permission of John Wiley & Sons from [61].

The FT-IR analysis performed allowed confirming the structure obtained—the disappearance of absorption bands originating from alkyne confirms the occurrence of 1,3-cycloaddition (Figure 12). In addition, adsorption bands originating from the triazole ring (3146 and 1589 cm${}^{-1}$) were observed on the spectrum.

**Figure 13:**
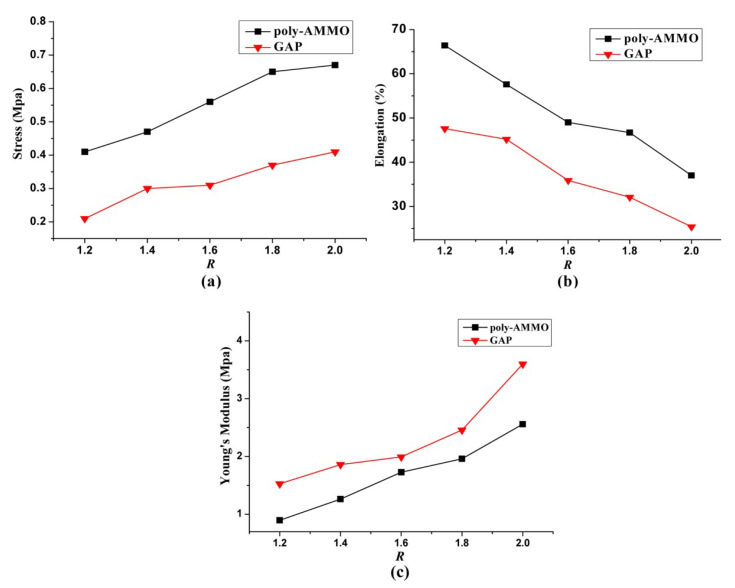
(**a**) Effect of R on the tensile strengths, (**b**) Effect of R on the elongation at break, (**c**) Effect of R on the Young’s Modulus. Reprinted with permission of John Wiley & Sons from [61].

Basic mechanical properties (the tensile strength, Young’s modulus and elongation at break) were determined for the obtained polymer samples (Figure 13). The analysis of the results made it possible to establish that as R increases, the cross-linking density increases, leading to an increase in stiffness and tensile strength. An increase in tensile strength was observed for GAP-based polymers from 0.21 to 0.41 MPa, and for poly(AMMO)-based polymers from 0.41 to 0.67 MPa. A similar correlation was observed for the Young’s Modulus—an increase was observed for GAP- and poly(AMMO)-based samples from 0.89 to 2.56 MPa and from 1.52 to 3.60 MPa, respectively. The mechanical strength of polymers based on poly(AMMO) was better than for polymers based on GAP, while for Young’s modulus an inverse relationship was observed.

Recently, the synthesis of carboxylated GAP copolymers has been described and were reported to exhibit improved thermomechanical properties (T_G_ = −48–−55 °C) in comparison with GAP (T_G_ = −49 °C), as well as a higher heat of combustion, while maintaining thermal decomposition temperature similar to that of GAP 228–230 °C, as compared with 227 °C for GAP) [62].

Copolymers of BAMO with carboxylated BAMO derivatives were similarly synthesised—20% of the poly(BAMO) chain was replaced with long-chain carboxylates—butanoate, octanoate and decanoate [63]. Carboxylated BAMO copolymers were synthesised by adding sodium azide and potassium carboxylate to poly(BCMO). Through a steric congestion resulting from the attachment of two azide groups to one carbon atom of poly(BAMO), the substitution of chloride atoms did not occur completely at 120 °C. For this reason, the synthesis of poly(BAMO-carboxylate) compounds was divided into two steps—substitution of poly(BCMO) with the corresponding carboxylate, and later addition of sodium azide. The substitution of chloride groups with azide groups was carried out for 48 h. The obtained compounds were characterised in terms of the typical properties of such substances. Compared to poly(BAMO), these compounds have lower glass transition temperatures, higher decomposition temperatures and lower sensitivity (Table 3). It was observed that as the alkyl chain increases, the glass transition temperature decreases.

**Figure 14:**
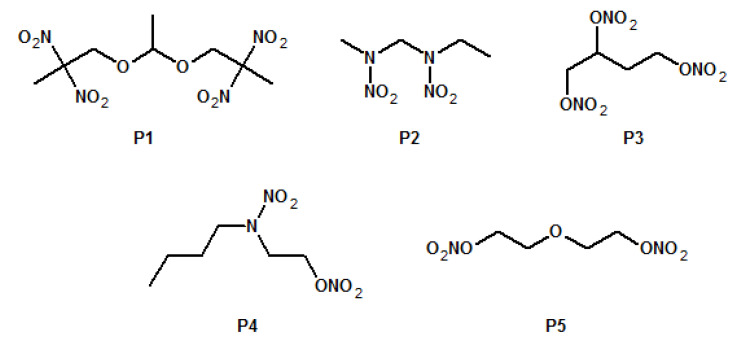
Chemical structures of energetic plasticising agents investigated in [64].

Another approach to modifying the glass transition temperature of energetic binders is to use plasticising agents, which themselves can also be energetic, as seen by a recent work on poly(NIMMO) modification [64]. A series of five different nitro- and nitrate-functionalised plasticising agents (P1-P5) were investigated (Figure 14). The plasticising agents appear to be compatible with poly(NIMMO), only minimally influencing its decomposition temperature and significantly lower the glass transition temperature of poly(NIMMO), with P4 and P5, shifting it to approx. −52 °C, highlighting the value of the two agents.

Novel hydroxy and nitrato telechelic oligomers of monomers bearing negatively inductive substituents, NIMMO and glycidyl nitrate, have been prepared by cationic polymerization method with BFFt as catalysator is presented in [39]. The activated monomer mechanism has been used to form the hydroxy oligomers, which have then been modified using dinitrogen pentoxide to form nitrate oligomers, without side reactions, e.g., chain scission. The nitrated oligomer has a lower T_G_, −32 °C, compared with the hydroxyl-terminated oligomer, −26 °C. According to the authors these materials are expected to act as energetic and fully compatible plasticisers.

## 5. Properties and Structure of Energetic Oxetane Polymers and Copolymers

### 5.1. Thermal Properties and Thermally-Induced Decomposition

The extensive study of the decomposition mechanism of poly(NIMMO) is an interesting insight into the properties of energetic oxetane polymers [22]. Not only has a mechanism for the thermally-induced decomposition been proposed, but clear evidence that decomposition of poly(NIMMO) takes place at temperatures as low as 129 °C is provided, undermining the values reported based on high-speed DSC measurements, which would indicate that the decomposition temperature of poly(NIMMO) is on the order of 180–220 °C [23].

Copolymers of energetic oxetanes (AMMO, NIMMO) with tetrahydrofuran have also been investigated in terms of their thermally-induced degradation [65]. Triblock copolymers were produced, with energetic oxetane and tetrahydrofuran repeat units constituting the two outer blocks and the middle block respectively. These copolymers were compared with polytetrahydrofuran, with the study finding the NIMMO-based triblock to be most thermally resistant, followed by the AMMO-based triblock and with polytetrahydrofuran being the least thermally stable. An initial insight into the mechanism of the degradation of these systems has also been proposed, but further investigation is required.

Triblock and random block copolymers of tetrahydrofuran with NIMMO, AMMO, BAMO and BEMO were also investigated by DSC [66]. NIMMO-based triblocks were found to exhibit decomposition at the lowest temperature among the tested copolymers, i.e., at 132°C, similar to the decomposition of poly(NIMMO) mentioned above. This is likely due to the significant length of the NIMMO blocks, constituting a separate phase, exhibiting poly(NIMMO)-like properties. This temperature was, however, increased significantly (186°C) after the triblock copolymer was cured via treatment with a diisocyanate and pentaerythritol. NIMMO-based polymers exhibited relatively higher decomposition enthalpies and less thermal stability than the others. The Authors have also correlated the copolymer chain topology with thermal properties, particularly with the glass transition temperature (T_G_), as shown in Table 4.

An experimental study of the thermal features of statistical copolymers of BAMO and NIMMO, as well as the parent homopolymers oddly reports the thermal decomposition temperatures of poly(NIMMO) and poly(BAMO) as 226 °C and 261 °C respectively, conflicting with the abovementioned results reported for poly(NIMMO) [40]. This, however, may be merely an experimental error, as the thermogravimetric measurements, on which the data were based, were conducted at a heating rate of 10 K/min, and are likely burdened by significant thermal inertia. Although the specific decomposition temperatures are likely unreliable, the thermal characteristics of the produced copolymers were a clearly distinguishable superposition of the features of the two parent homopolymers.

Copolymers of BAMO and AMMO were produced via treating poly(BAMO) and poly(AMMO) with diisocyanates, followed by coupling the resultant moieties using 1,4-butanediol, resulting in fairly complex structures, containing significant amounts of non-energetic segments [67]. The temperatures of the first decomposition step of the copolymers are on the order of 220°C and the kinetics of this step are investigated. The reported activation energies increase with increasing degree of conversion, indicating that a transition between at least two processes is taking place, with the higher activation energy process becoming more pronounced at higher degrees of conversion.

An interesting work reports the heats of formation and of combustion of poly(NIMMO), GAP, poly(GLYN), as well as energetic thermoplastic elastomers based on those polymers [68]. While the investigated systems differ only slightly in terms of their heats of combustion and these results are not accompanied by any other data (e.g., oxygen balance, specific impulse, compatibility with common propellant formulations), a significant discrepancy between heats of formation reported in literature is observed, indicating a need for an in-depth and rigorous study of this aspect.

### 5.2. Spectroscopic Investigation of Chemical Structures of Polyoxetane EBs

IR spectroscopy can be used to differentiate between poly(BAMO) and poly(AMMO), with several diagnostic signals being identified [69]. Interestingly, despite the use of wide range IR spectormetry, these signals are observed within the most common IR spectroscopic range (4500–500 cm^−1^).

An important aspect of working with new high-energy materials is to know their structure and relate it to their properties. Infrared spectroscopy techniques from a wide spectral band were used to describe the structure of compounds (NIR/MIR/FIR) [69].

Gas products of thermolysis of poly(glycidyl azide) (GAP), 3-azidomethyl-3-methylox-etane polymer (poly(AMMO)), 3,3-bis(azidomethyl)oxetane polymer (poly(BAMO)), 3-azidoxetane (AZOX) and 3-(2,3-diazidopro-poxymethyl)-3-methyloxetane (DAPMMO) were analysed [70].

It was noted that the main gas product of thermolysis of these polymers is HCN. The exception is poly(AMMO), where CO is predominantly emitted. Additional analysis using TG/FT-IR confirmed the decomposition process, with higher amounts of HCN released in the decomposition of poly(BAMO). It was also observed that for copolymers, the amount of HCN increased with increasing BAMO [71].

The spectra of poly(AMMO) and poly(BAMO) differ from those of their corresponding monomers primarily by the disappearance of the absorption band at 980 1/cm, which is due to the opening of the oxetane ring. In the region of 1000–1100 1/cm, absorption bands originating from C-O stretching bonds appear, and at 33,450 1/cm bands originating from the OH bond forming at the end of the polymer chain.

## 6. Applications

Low-vulnerability ammunition is a type of ammunition that, compared to classical ammunition, exhibits reduced sensitivity to thermal and mechanical initiating stimuli. One key application of poly(NIMMO) is in high-energy propellant formulations for low-vulnerability ammunition, as seen by the initial investigation of a poly(NIMMO)/HMX composite propellant [72]. Apart from low sensitivity to initiating stimuli, the propellant exhibits a significant impetus of 1230 kJ/kg.

In a recent theoretical study, block copolymers of BAMO and GAP were envisioned as the binder in hypothetical propellant formulations [73]. These formulations were composed of the copolymers, aluminium and ammonium perchlorate. The work included predicting the specific impulse of hypothetical formulations, in which ammonium perchlorate was partially replaced with HMX, RDX, CL-20 and ammonium dinitramide, however, no experimental results were reported to verify the validity of the predictions.

In an experimental study of a similar scope, statistical copolymers of BAMO and GAP were synthesised via copolymerisation of epichlorohydrin and bisbromomethyl oxetane, followed by azidation and were evaluated in prospective propellant formulations alongside GAP [74]. The propellants were either based on ammonium perchlorate or a mixture of ammonium perchlorate and RDX, with the formulations containing the investigated binders being cured using a standard isocyanate agent (Desmodur N100). The propellant formulations were designed to resemble the composition of an established composite propellant utilising HTPB as a binder (AP 1092) and found to have a higher burn rate than AP 1092 and to have a relatively low pressure exponent, being advantageous for some applications.

Propellants based on a GAP-BAMO copolymer and poly(AMMO), as energetic binders (EBs) were analysed in [75]. The investigated propellant formulation contained aluminium and ammonium perchlorate, as well as a binder. The reported new EBs were tested against HTPB in terms of performance in this formulation. The elongation at break in a stress-strain test was considerably lower than that obtained with HTPB, but the burning rate of the propellants based on EBs showed a pressure exponent significantly lower than for propellants based on HTPB. Interestingly, the burning rates of EB-based propellants were almost constant for pressures increasing in the range of 3–7 MPa.

## 7. Discussion and Future Outlook

Extensive work has been conducted on the synthesis of energetic polyoxetanes, as well as on the investigation of their properties and their application as EBs. These efforts have largely been focused on the development of systems with favourable mechanical properties, with a particular focus on glass transition temperature. Interestingly, although works dedicated to investigating the energetic parameters or performance of polyoxetane EBs in propellant formulations are present, they are relatively scarce. This trend has slowly been reversing in recent years, as significant strides were taken in terms of the investigation of the decomposition mechanisms of such compounds, however, such studies are largely limited to the binders themselves rather than including cured binders in any specific propellant formulation. Hence, a significant deficit of in-depth information on this aspect persists.

In terms of available results, the great variety of employed methods is insufficiently grounded in the use of reference materials and standardisation measures. This is exemplified by even fundamental parameters, such as glass transition temperature or decomposition temperature reported in literature being in some cases greatly divergent. In some cases this can be traced to departure from established protocols (e.g., compatibility testing via high-speed DSC), but in other cases, works reporting differing parameters appear highly credible. This may be a systemic indication that not all factors influencing the properties of the investigated EBs have yet been identified or that some of those factors have not been screened-off to a sufficient extent.

Regardless of their reliability, the reported results can rarely be directly compared with each other, as only a scarce handful of reports include comparison of the polyoxetane energetic binders (EBs) with reference materials (e.g., well-defined HTPB or other EBs) and even fewer works conduct rigorous comparisons of binder performance in specific propellant formulations [74,75].

Despite the need for resolving the abovementioned challenges, energetic polyoxetanes are a promising class of EBs, due to their facile synthesis, many avenues of structural modification and relatively high chemical stability. The many works reviewed herein provide evidence for the broad array of properties that can be achieved by these polymers and fine-tuned for the purpose of specific applications. This can be considered an excellent “proof of concept” for their use as energetic binders.

The next stage of research on energetic polyoxetanes should be focused on comprehensively investigating the factors influencing the properties of those polymers and describing in detail their relationships. In this aspect, even fundamental factors, such as molecular weight distribution of the polymers or polymer/copolymer composition and chain topology should be taken into consideration. This has been already achieved in some works [49], however, this approach should be a systemic one and the discussion of the impact of those parameters should not be limited only to thermomechanical properties of the EBs.

In regards to the above, works on the development of plasticising agents and other additives to energetic polyoxetanes may appear to be premature, but in fact are of essential importance, as not only do they facilitate broadening the scope of material properties that may be achieved, but also provide a valuable insight into the interactions of energetic polyoxetanes with other chemical species.

Conversely, the importance of additives should not be over-estimated and focus should be maintained on the fundamental purpose of EBs - to promote cohesion between the components of energetic material formulations. As such, the interactions of energetic polyoxetanes and their compatibility with other components of such formulations should remain among the main foci of the postulated next stage of research on those promising materials.

## Figures and Tables

**Table 1:** Parameters detailing the composition of BAMO-AMMO copolymers reported in [30], as well as their fundamental thermal and mechanical properties.

**Molar Ratio (BAMO:AMMO)**	**Molecular Weight (Mn; Mw)**	**Dispersity Index**	**Hydroxyl Value [mg KOH]**	**T_G_ [°C]**	**Melting Point [°C]**
80:20	3431; 5200	1.5	32	−36	56
50:50	1200; 1600	1.3	93	−43	41
20:80	3064; 4475	1.46	36	−51	9
**Molar Ratio (BAMO:AMMO)**	**T_MEASURE_ [°C]**	**Storage Modulus [Pa]**		**Crossover Frequency [Hz]**	**Viscosity [cPs]**
80:20	75	2.6 × 10^12^		17	1000 (solid at RT)
50:50	50	8.8 × 10^13^		7	20,000
20:80	30	3.73 × 10^13^		17	10,400

**Table 2:** Comparison of mechanical properties of BAMO-AMMO copolymers cross-linked with common diisocyanate agents investigated in [46].

Utilised Diisocyanate	σ_m_ [MPa]	ε_b_ [%]
HDI	4.04	289
TDI	5.24	390
IPDI	4.96	375
HMDI	4.71	312

**Table 3:** Parameters detailing the composition of poly(BAMO) and carboxylated BAMO copolymers reported in [63], as well as their fundamental thermal properties.

Polymer/Copolymer	K_2_CO_3_:NaN_3_ [% mol.]	Yield	M_W_ [g/mol]	M_N_ [g/mol]	Dispersity Index	T_G_ [°C]	T_DEC_ [°C]
poly(BAMO)	-	-	2460	2030	1.21	−37	211
Poly(BAMO-butyrate)	80:20	92	2720	2080	1.30	−43	233
Poly(BAMO-octanoate)	80:20	90	2910	2390	1.22	−47	237
Poly(BAMO-decanoate)	80:20	91	2980	2440	1.22	−51	237

**Table 4:** Glass transition (T_G_) and decomposition temperatures (T_DEC_) of random and block copolymers of THF and selected energetic oxetanes. Reprinted with permission of John Wiley & Sons from [66].

Co-Monomer	T_G_ RC ^1^ [°C]	T_G_ BC ^2^ [°C]		Polymer T_DEC_^3^ [°C]	Monomer T_DEC_^3^ [°C]
THF	−75	-	-	-	-
BAMO	−23	−76	−8	242	160
AMMO	−41	−70	−32	202	139
NIMMO	−28	−68	−19	132	113
BEMO	−44	−72	−40	-	-

^1^ RC—random copolymers; ^2^ RC—block copolymers; ^3^ TDEC—temperature of decomposition based on high-speed thermogravimmentry.

## Data Availability

Not applicable.

## Abbreviations

The following abbreviations are used in this manuscript:

GAP	Poly(glycidyl azide)
HTPB	Hydroxyl-terminated polybutadiene
P(4-VP)	Poly(4-vinylpyridinium)
PAA	Poly(allyl azide)
PBAN	Butadiene-acrylonitrile-acrylic acid terpolymer
PE	Polyethylene
PEO	Poly(ethylene oxide)
PMMA	Poly(methyl methacrylate)
Poly(GLYN)	Poly(glycidyl nitrate)
PVC	Poly(vinyl chloride)
PVT	Poly(vinyl tetrazole)
PS	Polystyrene
